# Taurine alleviates endoplasmic reticulum stress in the chondrocytes from patients with osteoarthritis

**DOI:** 10.1080/13510002.2018.1445581

**Published:** 2018-03-01

**Authors:** Yiqun Bian, Hao Wang, Shui Sun

**Affiliations:** a Shandong University, Jinan, People's Republic of China; b Liaocheng People’s Hospital, Liaocheng, People's Republic of China; c Hospital of Traditional Chinese Medicine of Liaocheng City, Liaocheng, People's Republic of China; d Shandong Provincial Hospital, Jinan, People's Republic of China

**Keywords:** Taurine, ER stress, osteoarthritis, H_2_O_2_, anti-apoptosis

## Abstract

Osteoarthritis (OA), characterized by pain and stiffness, swelling, deformity and dysfunction of joints, affects large numbers of population. The purpose of this study was to discover the effects of taurine in human OA chondrocytes and explore the underlying mechanisms. 46 patients with different grades of OA were recruited. Of these patients, 24 underwent total knee replacement and cartilages were harvested. The mRNA expressions of type II collagen (Collagen II) and endoplasmic reticulum (ER) stress markers (GRP78, GADD153 and Caspase-12) in cartilages were quantified by qRT-PCR. Cell viability and apoptosis of patient-derived chondrocytes were assessed by the CCK-8 assay and flow cytometry assay, respectively. Meanwhile, protein levels of Collagen II and ER stress markers both in cartilages and chondrocytes were evaluated by Western blot. The mRNA and protein levels of Collagen II decreased as OA progressed, while the expressions of ER stress markers increased dramatically. H_2_O_2_ induced ER stress in chondrocytes, as shown by the significant increase in the expression of ER stress markers, inhibited chondrocyte viability and Collagen II synthesis, promoted apoptosis. However, taurine treatment inhibited these above phenomena. These results indicated that taurine exhibited anti-OA effect by alleviating H_2_O_2_ induced ER stress and subsequently inhibiting chondrocyte apoptosis.

## Introduction

Osteoarthritis (OA), a common chronic disease that affects the joints, can be caused by aging, mechanical injury, overweight, obesity and impairment of peripheral nerves [1,2]. The clinical manifestations of OA contain articular cartilage degradation and subchondral bone sclerosis, which may lead to joint stiffness, deformity and dysfunction [3]. Chondrocytes, the only cells existing in articular cartilage, can generate and maintain the articular cartilaginous matrix, which is composed mainly of collagen and proteoglycans [4]. Recent reports have demonstrated that elevated chondrocyte loss caused by apoptosis is a major feature of OA [5,6].

Endoplasmic reticulum (ER) stress, which occurs due to an imbalance between the load of unfolded or misfolded proteins in the ER and the processing capacity of ER, participates in many disease pathologies [6,7]. Recent studies have demonstrated that ER stress in chondrocytes is responsible for chondrocyte apoptosis along with the progression of OA [4,8,9].

Taurine, first isolated and characterized from the bile of the ox, is one of the most abundant endogenous free amino acids in humans [10]. It has been implicated in several essential biological processes including bile acid conjugation, calcium modulation, osmoregulation, membrane stabilization and protein phosphorylation. Moreover, anti-apoptosis and anti-oxidant properties are essential for the cytoprotective functions of taurine [11,12]. Previous studies have confirmed that taurine inhibits ER stress-induced apoptosis and protects against lung injury, stroke and neurodegenerative diseases [11,13].

However, no study has been done to examine the possible protective functions of taurine on human OA yet. Therefore, we made a hypothesis that taurine treatment might protect against OA by attenuating ER stress-associated apoptosis. To identify that, cartilages were isolated from 24 OA patients who received total knee replacement. The mRNA and protein levels of type II collagen (Collagen II), glucose-regulated protein 78 (GRP78), growth arrest and DNA-damage inducible gene 153 (GADD153) and Caspase-12 in cartilages from patients with different OA grades were quantified by qRT-PCR and Western blot analysis, respectively. OA patient-derived chondrocytes were cultured in three conditions including: No treatment (Control group), H_2_O_2_ treatment to induce ER stress (H_2_O_2_ group) and preincubation with taurine before H_2_O_2_ exposure (H_2_O_2_ + taurine group). The viability and apoptosis of cultured human OA chondrocytes were assessed by the CCK-8 assay and flow cytometry assay, respectively. Meanwhile, Western blot was also employed to evaluate the protein levels of Collagen II and ER stress markers in chondrocytes with different treatments. Our results illustrated that ER stress is highly involved in the H_2_O_2_-induced apoptosis in chondrocytes. Moreover, these results for the first time established that taurine alleviated ER stress in human OA chondrocytes, as shown by the significant decrease in the expressions of ER stress markers, promoted chondrocyte viability and Collagen II synthesis, and inhibited chondrocyte apoptosis.

## Methods & materials

### Ethical considerations

All experiments and procedures were reviewed and approved by the institutional ethical review board of Liaocheng People’s Hospital, China. All the participants were informed of the purpose of this study and provided informed written consent.

### Patients

**Figure 1.**
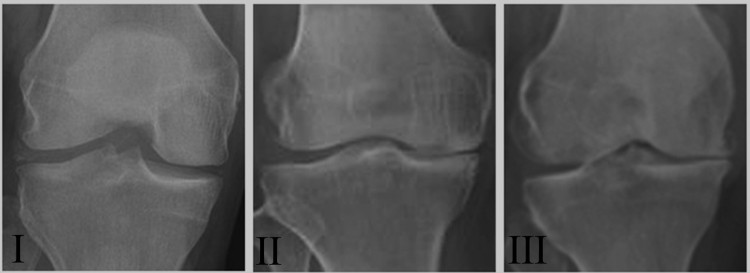
Representative radiographs of patients with different OA grades. Radiographs II (*n* = 22) and III (*n* = 15) demonstrated the degenerative changes of OA while radiographs I (*n* = 9) illustrated a preserved joint space.

Based on the American College of Rheumatology criteria, a total of 46 patients diagnosed with OA in the Department of Orthopaedic Surgery at the Liaocheng People’s Hospital between February 2012 and May 2017 (*n* = 46, 23 females and 23 males) were enrolled in this study. Inclusion criteria specified men and non-pregnant women, age 18–70 years, with primary OA of at least one knee. Primary OA was defined by deterioration and abrasion of articular cartilage (joint space narrowing) or formation of new bone (osteophytes) at the joint surface of the knee (medial tibio-femoral, lateral tibio-femoral or patello-femoral), demonstrated on a radiological examination carried out within the previous 3 months. Knees of OA patients were examined by clinical and radiological evaluations and subsequently subdivided into 3 groups (grades I, II and III) according to the degree of cartilage degeneration based on the Kellgren–Lawrence radiographic grading scale (Figure 1) [14]. Of these patients, 24 underwent total knee replacement and articular cartilage samples of them were collected (*n* = 24, 12 females and 12 males, aged between 18 and 65 years).

The following exclusion criteria was applied to the entire cohort: rheumatoid arthritis (RA) or other inflammatory arthritis (including psoriatic arthritis, post-infectious arthritis and metabolic arthritis, traumatic arthritis or surgical joint replacement), defined as self-report of a physician diagnosis; corticosteroid use or ever use of any RA-specific prescription medications; unlikely to demonstrate measurable loss of joints space during the study, defined as severe joint space narrowing on the baseline fixed flexion knees radiograph; hepatic or peptic ulcer disease; history of alcohol or drug abuse; lactation; concomitant skin disease at the application site; fibromyalgia; other painful or disabling condition affecting the knee; other serious diseases, such as cancer, stroke, and so on.

### Specimen processing

Harvested cartilages were rinsed in PBS. In a sterile state, several frozen cartilages of grade I, II and III were separately minced into small pieces. Pieces of the tissues were used for protein extraction and total RNA extraction, and the remaining samples were kept at −70°C.

### OA patient-derived chondrocyte culture

Following washing with PBS, harvested cartilage from 6 OA patients of grade II was cut into small pieces and digested using 0.2% collagenase (Sigma, MO, USA) at 37°C overnight. After digestion, the chondrocytes were collected by a 200 µm filter, and cultured in Dulbecco’s modified Eagle’s medium (DMEM; Gibco, Gaithersburg, USA) supplemented with 10% fetal bovine serum (FBS; Invitrogen, Carlsbad, CA, USA), 100 U/ml penicillin, and 100 μg/ml streptomycin. Chondrocytes were cultured in monolayer (2 × 10^6^ cells/well in 12 well plates) at 37°C in a 5% CO_2_ incubator and the medium were changed every 3 days.

When cell confluency was close to 90%, the adherent chondrocytes were passaged following trypsinization (0.25% Trypsin-EDTA, 2-3 mins). Then the cells were collected following the second passage and seeded at a density of 0.6 × 10^6^ cells per well in 6 well plates. The chondrocytes were subdivided into 3 groups: Control group (no treatment), H_2_O_2_ group (treatment with 0.3 mM H_2_O_2_ for 4 h on day 4 after seeding), and H_2_O_2_ + taurine group (treatment with 25 mM taurine for 24 h prior to H_2_O_2_ exposure).

### Western blot analysis

To analyze the mechanisms of taurine in the treatment of OA, Western blot was performed as previously described [15]. Cells or cartilages, which were frozen and ground to fine powder in liquid nitrogen, were lysed in RIPA buffer containing 1% (v/v) mammalian protease inhibitor. Following lysate clarification, protein concentrations were determined. Subsequently, soluble lysates were boiled in 2% SDS sample buffer for 5 min. Equivalent amounts of protein were separated on SDS-PAGE and electroblotted onto a nitrocellulose membrane (Pierce, WI, USA). After blocking, membranes were incubated with goat polyclonal antibodies against Collagen II (1:500; Proteintech), GRP78 (1:1000; Abcam, Cambridge, MA, USA), GADD153 (1:500; Cell Signaling Technology, Danvers, MA, USA) and Caspase-12 (1:2000; Cell Signaling Technology, USA). Following extensive washes, the membranes were incubated with HRP-conjugated anti-goat secondary antibodies (1:3000; Abcam, USA). The protein immuno- complex was visualized and analyzed (relative to β-actin expression) using an ECL system (Pierce, USA).

### RNA extraction and real-time quantitative PCR (qRT-PCR) analysis

Total RNA was prepared from patient cartilages using the TRIzol Reagent (Invitrogen, USA) according to the manufacturer’s instructions. SYBR® Premix Ex Taq™ II kit (TaKaRa, shanghai, China) was used to quantify the expression levels of Collagen II, GRP78, GADD153 and Caspase-12 according to the protocol provided. Relative expression of above genes was measured by the 2^−ΔΔCt^ method. The first ΔCT was the difference in threshold cycle between the target and reference genes, and the ΔΔCT was the difference in ΔCT as described in the above between the target and reference samples, which was ΔΔCT = ΔCT(a target sample) − ΔCT(a reference sample). The final result of this method was presented as the fold change of target gene expression in a target sample relative to a reference sample, normalized to a reference gene. In this study, glyceraldehyde-3-phosphate dehydrogenase (GAPDH) was used as reference gene. All reactions were performed in triplicate. All primers were listed as below:

Collagen II

Forward 5′-CCA CAC TCA ATC CCT CAA C-3′

Reverse 5′-GCT GCT CCA CCA GTT CTT C-3′;

GRP78

Forward 5′-TCC TAT GTC GCC TTC ACT-3′

Reverse 5′-ACA GAC GGG TCA TTC CAC-3′;

GADD153

Forward 5′-CTG ACC AGG GAA GTA GAG G-3′

Reverse 5′-TGC GTA TGT GGG ATT GAG-3′;

Caspase-12

Forward 5′-AAT CTG TGG GAC CAA GCA-3′

Reverse 5′-GAG CCT TTG TAA CAG CAT CA-3′;

GAPDH

Forward 5′-ACC CAG AAG ACT GTG GAC TT-3′

Reverse 5′-TTC TAG ACG GCA GGT CAG GT-3′.

### CCK-8 assay

Cell viability and number was analyzed using CCK-8 solution (Promega, Beijing, China) in accordance with the manufacturer’s instructions. Cells (1 × 10^4^/well) were seeded in three replicate wells on a 96-well plate and treated with 20 μL/well of CCK-8 solution for 4 h at 37°C. The absorbance at 490 nm was measured via a microplate reader (Bio-Rad, Hercules, CA, USA). All reactions were performed in triplicate.

### Flow cytometry assay

Annexin V-FITC/propidium iodide (PI) double staining was used to quantify apoptosis. In brief, chondrocytes were collected and stained with Annexin V-FITC solution and PI in the dark for 15 min at room temperature. Subsequently, cells were washed and resuspended in binding buffer. Stained cells were analyzed by FACScan flow cytometer (BD Biosciences, Franklin Lakes, NJ, USA). Cells incubated with binding buffer alone were set as negative control samples.

### Statistical analysis

The data are presented as the means ± SEM. One-way ANOVA followed by a Tukey’s post hoc test was used to analyze significant differences between treatment groups. All the statistical analyses were performed by SPSS software 13.0 and statistical significance was defined as a *p*-value < 0.05, <0.01 or <0.001.

## Results

### The expressions of Collagen II and ER stress markers in the cartilage of OA patients associated with the severity of OA progression

**Figure 2.**
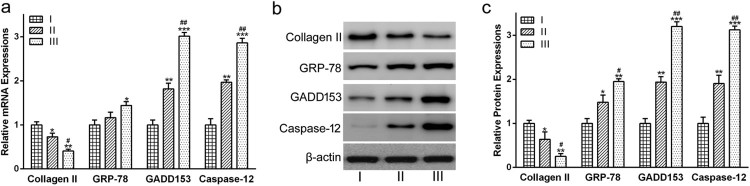
The expression of Collagen II, GRP78, GADD153 and Caspase-12 in cartilages from patients with different OA grades. (a) mRNA and (b) protein expressions of Collagen II and ER stress markers were determined via qRT-PCR analysis and Western blot analysis, respectively. (c) was the statistical analysis of (b). Data were presented as mean ± SEM. Experiments were repeated in triplicate. *p < 0.05, **p < 0.01 and ***p < 0.001 compared to grade I. #*p* < 0.05 and ##*p* < 0.01compared to grade II.

Compared with grade I, the levels of Collagen II mRNA were decreased significantly in grades II (Figure 2(a), *p* < 0.05) and III (Figure 2(a), *p* < 0.01). In contrast, the mRNA levels of GADD153 and Caspase-12 were increased dramatically in grades II (Figure 2(a), *p* < 0.01) and III (Figure 2(a), *p* < 0.001), as compared with grade I. Moreover, both GADD153 and Caspase-12 mRNA levels in grade III were significantly higher than those in grade II (Figure 2(a), *p* < 0.01). There was no significant difference between grades I and II in GRP78 mRNA expression. However, the GRP78 mRNA level increased obviously in grade III compared with that in grade I (Figure 2(a), *p* < 0.05).

As for gene expression, Collagen II protein levels were decreased dramatically in grades II (Figure 2(b) and (c), *p* < 0.05) and III (Figure 2(b) and (c), *p* < 0.01), as compared to grade I. Moreover, Collagen II protein level in grade III was significantly lower than in grade II (Figure 2(b) and (c), *p* < 0.05). In the case of GADD153 and Caspase-12, protein expression levels were increased dramatically in grades II (Figure 2(b) and (c), *p* < 0.01) and III (Figure 2(b) and (c), *p* < 0.001), as compared with grade I. Moreover, both GADD153 and Caspase-12 protein levels in grade III were dramatically higher than those in grade II (Figure 2(b) and (c), p < 0.01). Figure 2b and c also indicated that the GRP78 protein abundance increased gradually with the severity of OA.

### 0.3 mM H_2_O_2_ and 25 mM taurine were chosen for the subsequent experiments

**Figure 3.**
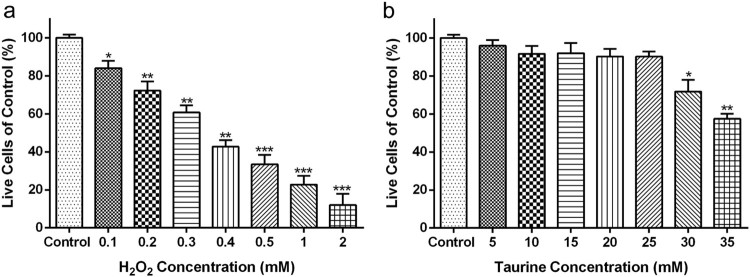
Concentration dependent toxicity of taurine and H_2_O_2_. Effects of (a) H_2_O_2_ (0.1, 0.2, 0.3, 0.4, 0.5, 1 and 2 mM) and (b) taurine (5, 10, 15, 20, 25, 30, 35 mM) on the cell viability of OA patient-derived chondrocytes were measured via CCK-8 assay. Data were presented as mean ± SEM. *p < 0.05, **p < 0.01 and ***p < 0.001 compared to control.

The chondrocytes from OA patients were treated with H_2_O_2_ at various concentrations (0–2 mM) for 4 h. The results showed that the survival rate of chondrocytes decreased with the increasing of H_2_O_2_ concentrations (Figure 3(a)), which indicated that H_2_O_2_ administration decreased the chondrocyte viability in a dose-dependent manner. Compared with the control group, exposure to 0.3 mM H_2_O_2_ for 4 h led to dramatic cytotoxicity and approximately 60% of the chondrocytes remained viable (Figure 3(a), *p* < 0.01). At 0.4, 0.5, 1 and 2 mM H_2_O_2_ for 4 h, the survival rate of chondrocytes decreased to 42%, 33%, 23% and 11%, respectively, compared with the control group (Figure 3(a)). Thus, 0.3 mM H_2_O_2_ was chosen for the subsequent experiments.

Chondrocytes were treated with 0–35 mM taurine for 24 h, and the data showed that 0–25 mM taurine had no apparent effect on chondrocyte viability (Figure 3(b)), while 30 mM (Figure 3(b), *p* < 0.05) or 35 mM (Figure 3(b), *p* < 0.01) taurine resulted in significant reduction in the survival percentage of chondrocytes. Thus, 25 mM taurine was chosen for the subsequent experiments.

### Taurine protected chondrocytes against damage induced by H_2_O_2_ in vitro

**Figure 4.**
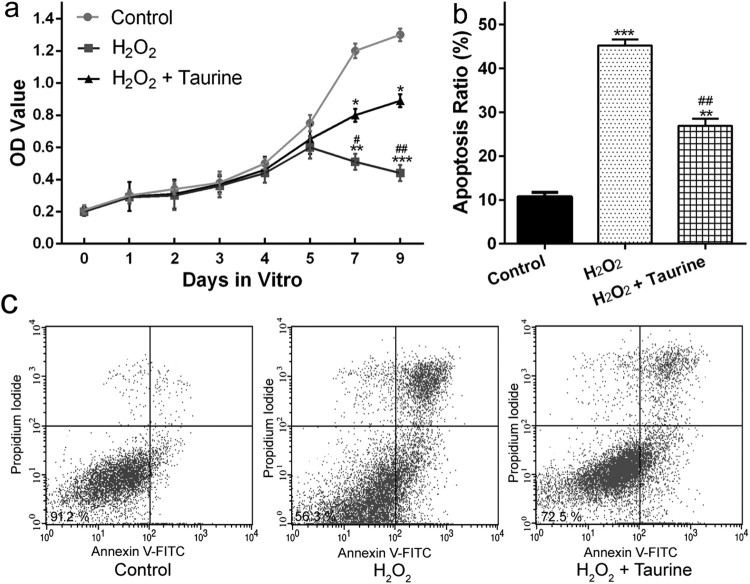
Taurine treatment affected the viability and apoptosis of H_2_O_2_-stimulated chondrocytes. (a) chondrocyte viability was measured by the CCK-8 assay. (b), statistical bar graph showing the apoptosis ratio. (c), chondrocytes were stained with Annexin V/propidium iodide and analyzed by ﬂow cytometry. Data were presented as mean ± SEM. Experiments were repeated in triplicate. *p < 0.05, **p < 0.01 and ***p < 0.001 compared to control. #*p* < 0.05 and ##*p* < 0.01compared to the H_2_O_2_ group.

To examine the effects of taurine on H_2_O_2_-stimulated chondrocytes, CCK-8 assay and Annexin V-FITC/PI double binding assay were used to measure the viability and apoptosis of chondrocytes, respectively. As shown in Figure 4(a), H_2_O_2_-induced oxidative stress in chondrocytes resulted in a markable reduction in cell viability at day 7 (*p* < 0.01 or *p* < 0.001). The impaired cell viability was significantly recovered by taurine treatment (*p* < 0.05) (Figure 4(a)).

As for cell apoptosis (Figure 4(b) and (c)), showed that the apoptotic cell number of the H_2_O_2_ + taurine group was significantly lower than that of the H_2_O_2_ group, which indicated that preincubation with taurine had a protective effect by reducing or inhibiting the apoptosis of H_2_O_2_-treated chondrocytes.

### Taurine inhibited ER stress induced by H_2_O_2_ stimulation in human OA chondrocytes

**Figure 5.**
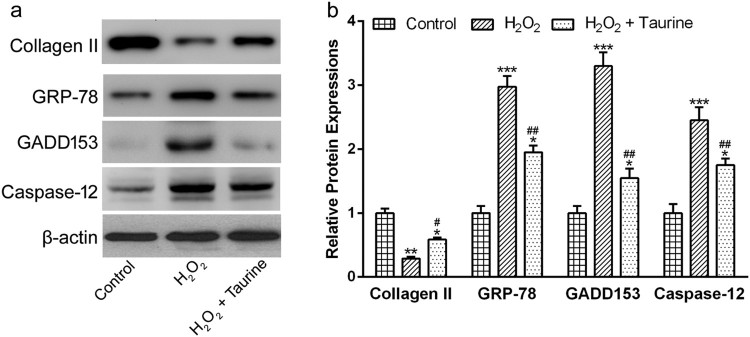
Taurine treatment affected the expressions of Collagen II, GRP78, GADD153 and Caspase-12 in H_2_O_2_-treated chondrocytes. (a)Western blot were used to assay the protein expressions and β-actin was used as a loading control. (b) was the statistical analysis of (a). Data were presented as mean ± SEM. Experiments were repeated in triplicate. *p < 0.05, **p < 0.01 and ***p < 0.001 compared to control. #*p* < 0.05 and ##*p* < 0.01 compared to the H_2_O_2_ group.

To explore the inhibitory effects of taurine on ER stress-mediated apoptosis, we measured the protein levels of Collagen II and three ER stress markers. Under normal condition, there were lots of Collagen II in the chondrocytes. H_2_O_2_ stimulation dramatically decreased the protein abundance of Collagen II by nearly 73% (Figure 5(a) and (b), *p* < 0.01), compared to the control group. However, taurine treatment significantly increased the abundance of Collagen II by 100% (Figure 5(a) and (b), *p* < 0.05), compared to the H_2_O_2_ group.

The results of Western blot also showed that GRP78, GADD153 and Caspase-12 were hardly expressed in human OA chondrocytes under normal conditions (Figure 5(a) and (b)). The stimulation of H_2_O_2_ significantly enhanced the expressions of all three ER stress markers (Figure 5(a) and (b), *p* < 0.001). Interestingly, taurine treatment significantly inhibited these phenomena (Figure 5(a) and (b), *p* < 0.01).

## Discussion

OA, caused by abnormal mechanical stress loaded on the cartilages and low level inflammatory processes, is one of the serious concerns to the health of human beings and will ultimately result in joint degeneration, or even disability [16]. Previous studies have indicated that elevated chondrocyte apoptosis, which is associated with the degradation of cartilage matrix, is the hallmark of OA and chondrocyte apoptosis inhibition is vital for the treatment of OA [17,18].

ER, a key organelle, participates in the processes of protein synthesis, modification and secretion and is vital for cell survival and function [19,20]. Perturbations of intracellular calcium homeostasis cause the accumulation of misfolded and/or unfolded proteins in the ER, leading to a state known as ER stress. ER stress has been reported to be involved in ischemia reperfusion, diabetes mellitus, liver disease, kidney disease and neuro- degenerative disease [13,21–24]. In addition, a large number of studies have proved that ER stress is also associated with chondrocyte death by apoptosis *in vivo* and *in vitro* [6,14].

In this study, a total of 46 OA patients were recruited and patient cartilages were collected from 24 patients undergoing total knee replacement surgery. The mRNA and protein expressions of Collagen II and ER markers, such as GRP78, GADD153 and Caspase-12, were detected by qRT-PCR analysis and Western blot analysis, respectively. X-ray of grade III cartilage showed cartilage disappearance and marked joint space narrowing (Figure 1), and the mRNA and protein levels of Collagen II gradually decreased as OA progressed. Furthermore, the mRNA and protein expressions of the above ER markers, which were specific for ER stress, increased dramatically as cartilage degeneration worsened. Thus, the above results indicated that ER stress played a crucial role in the development of OA, which was consisted with previous reports [15].

Taurine, one of the abundant amino acids existing in mammalian tissues, can resist various types of damages by its anti-oxidant and anti-apoptosis activities in both clinical trials and animal studies [25–28]. Taurine could not only promote cell growth and maintain phenotype of human articular chondrocytes [29], but also ameliorate ROS-induced cartilage damage through its antioxidant property [15,30]. It is worth noting that a significant increase in NRF2 mRNA levels in the H_2_O_2_-stimulated chondrocyte is observed after treatment with taurine at a low concentration of 200 μM [31]. Whether such transcriptional regulation is lying behind alleviation of ER stress needs further research .Another thing needs more concern is the dose variability, which is relative common in clinical practice. The dose varies from 120 to 480 μM [31,32], while in our study the concentration of 25 mM taurine is the critical concentration which will do no apparent harm to chondrocyte viability. Such concentration is chosen to show the best characteristics its alleviation to ER stress.

It is known that an increase in Reactive oxygen species (ROS), including hydrogen peroxide (H_2_O_2_), superoxide (${\text{O}}_{2}^{-}$) and hydroxyl radical (·OH), impairs intracellular calcium homeostasis and subsequently results in ER stress [33]. Pan et al. (2010) found that H_2_O_2_ increased oxidative stress and up-regulated the expressions of GRP-78 and GADD153 in rat phenocromocytoma PC12 cells [34]. Banerjee et al. (2017) reported that that andrographolide treatment induced ROS production in colon cancer cells and that elevated ROS caused ER stress, which subsequently inducing cell apoptosis process [33]. In order to examine whether taurine exerted cytoprotective effects against H_2_O_2_ induced oxidative stress related to ER stress, Western blot analysis was employed to assay the protein levels of Collagen II and three ER stress markers. According to the results, H_2_O_2_ stimulation significantly enhanced the protein abundances of ER stress markers, which indicated the induction of ER stress by H_2_O_2_-stimulated oxidative stress, and taurine administration significantly inhibited these phenomena. In addition, taurine administration also prevented the decrease in Collagen II protein level by H_2_O_2_ treatment. Taken together, the above data provided compelling evidence that taurine had cytoprotective effects against H_2_O_2_ induced ER stress by promoting chondrocyte viability and inhibiting apoptosis.

In conclusion, our study demonstrated that the use of taurine had anti-apoptotic effects on OA patient-derived chondrocytes stimulated with H_2_O_2_. Taurine treatment promoted chondrocyte viability and inhibited chondrocyte apoptosis by suppressing the ER stress pathway, as evidenced by the up-regulation of the expression of Collagen II and the down-regulation of the expressions of ER stress markers. Thus, our results illustrated that taurine was a promising OA therapeutic agent. In the other hand, it is well-known that ER stress will further lead to the Unfolded Protein Response (UPR) in three major UPR signal transduction pathways, including ATF6, PERK and IRE1 signaling pathways [34]. Hence, further studies are still required to explore in detail which specific signaling pathway in ER was affected by taurine treatment. Additionally, the question of how H_2_O_2_-induced oxidative stress triggered ER stress remained unanswered and further experiments are also needed to elucidate the precise mechanisms responsible for the oxidative stress-elicited ER stress both *in vitro* and *in vivo*.

## Conclusion

This study provided solid evidence that taurine treatment exhibited anti-OA roles by suppressing H_2_O_2_-induced apoptosis in cultured chondrocytes of OA patients. The possible mechanism was that preincubation with 25 mM taurine alleviated H_2_O_2_-induced ER stress in chondrocytes by significantly inhibiting the expression of three ER stress markers and increasing Collagen II synthesis. Thus, our study showed that taurine was a promising OA therapeutic agent.
